# Asian Americans and infertility: genetic susceptibilities, sociocultural stigma, and access to care

**DOI:** 10.1016/j.xfre.2021.12.004

**Published:** 2021-12-18

**Authors:** Michelle H. Vu, Anh-Tho Antoinette Nguyen, Snigdha Alur-Gupta

**Affiliations:** Department of Obstetrics and Gynecology, University of Rochester Medical Center, Rochester, New York

**Keywords:** Access to care, Asian American, infertility

## Abstract

Infertility affects over 6 million people in the United States and has been shown to disproportionally affect minority patient populations. Asian American infertility is a particularly understudied area of research. This mini review article explores the current state of published research focusing on Asian American infertility trends as well as their barriers to fertility care. A small number of published studies have found that Asian American patients have decreased success with fertility treatments, including lower rates of pregnancy and live birth. These trends may be attributed to a combination of genetic, environmental, and cultural factors, which will be discussed here in further detail. It is crucial to continue building on Asian American fertility research to provide this diverse patient population with comprehensive, compassionate, and culturally sensitive care.

**Discuss:** You can discuss this article with its authors and other readers at https://www.fertstertdialog.com/posts/xfre-d-21-00148

**Essential Points**

• There are only a few studies investigating Asian American infertility. These studies report that Asian American patients have greater difficulty conceiving, wait longer to seek infertility treatment, and have decreased treatment success rates.
• Asian Americans are genetically and environmentally predisposed to infertility; however, cultural factors affect their willingness to seek fertility treatment.
• Recommendations for Asian American infertility research include differentiating between Asian racial and ethnic groups and increasing participation in research through the destigmatization of infertility.

Infertility affects >6 × 10^6^ women in the United States (1). In recent years, there has been an increase in publications demonstrating that infertility is more prevalent in minority patients than in White women (2). Despite the increased prevalence, studies have shown that minority women undergoing infertility treatment are less likely to be successful. The odds of pregnancy are reduced for Asians (odds ratio [OR], 0.86; 95% confidence interval [CI], 0.80–0.93), and the odds of live birth are reduced for all minority groups: Asian (OR, 0.90; 95% CI, 0.82–0.97), Black (OR, 0.62; 95% CI, 0.56–0.68), and Hispanic (OR, 0.87; 95% CI, 0.79–0.96) women in comparison to White women (3, 4). Asian American infertility and its treatment are particularly understudied areas of research. However, with increasing awareness about the role of ethnic disparities in reproductive outcomes, this subject has come to the forefront.

Asian is a race category encompassing a large contingent of people, including those with origins in the Far East, Southeast Asia, and the Indian subcontinent (5). According to the 2019 US Census Bureau population estimate, there are 18.9 × 10^6^ Asian Americans, accounting for 5.7% of the nation’s population (6). Despite the millions of Asian Americans seeking healthcare in our country, Asian Americans are underrepresented in the infertility literature. In a literature review evaluating infertility in the Asian race within the United States, only 10 studies were found for the past 15 years.

## Asian American infertility evaluation and management

Although there is self-reported data among US Black and Hispanic women showing an increase in infertility rates, no data currently exist on the self-reported rates of infertility in the Asian population (7). Data published in 2019 by the Centers for Disease Control and Prevention did note that Asian women had a lower number of births per 1,000 women of 1,511 than White women of 1,610.5 (8).

Asian American patients undergoing fertility treatment differ from their White counterparts in a few key baseline characteristics. The maternal age at which they begin to seek treatment is higher (34.7 ± 4.54 years vs. 33.7 ± 4.52 years for White women, *P*<.01) (9). Asian women are more likely to be nulligravid (58.9% vs. 52.9% for White women, *P*<.01) or nulliparous (85.2% vs. 78.1% for White women, *P*<.01). They are also more often given the diagnosis of diminished ovarian reserve (11.4% vs. 7.9% for White women, *P*<.0001) (4, 9). Not surprisingly, this has translated to poorer outcomes.

Asian American patients have decreased clinical pregnancy and live birth rates, even after receiving treatment. In a study conducted by Purcell et al. (9), the investigators studied two data sets: the Society for Assisted Reproductive Technology (SART) database from 1999 to 2000 and the data from patients attending the reproductive health clinic at the University of California, San Francisco, from 2001 to 2003. The study sample size included 25,843 White and 1,429 Asian patients from the SART database and 370 Caucasian and 197 Asian patients from the site-specific clinic. In the SART data set, the adjusted odds ratio (aOR) of having a live birth in Asian patients compared with White patients was 0.76 (95% CI, 0.66–0.88), and in the site-specific data set, the aOR was 0.59 (95% CI, 0.37–0.94). Multivariate analysis of this study concluded that Asian ethnicity itself was found to be an independent predictor of poor in vitro fertilization (IVF) outcomes (9). A subsequent study using the SART database from 2004 to 2006 published by Fujimoto et al. (4) echoed these findings. They studied 107,484 White patients and 13,671 Asian patients, reporting a lower OR of pregnancy in Asian patients than in White patients (OR, 0.86; 95% CI, 0.80–0.93) and of live birth (OR, 0.90; 95% CI, 0.82–0.97) (4). Baker et al. (10) also conducted a study using the SART database for the same time period of 2004–2006. The investigators reverberated the findings of the study by Fujimoto et al. (4); however, they also reported that Asians were more likely to experience stillbirth (0.8 vs. 0.5, *P*<.0001) and fetal loss (17.8 vs. 15.7, *P*<.0001) across all gestational ages (10). The most recent study using the SART database to examine ethnic disparities in infertility treatment published by Shapiro et al. (11) investigated assisted reproductive technology (ART) cycles, specifically 515,263 cycles in non-Hispanic White patients and 87,845 cycles in Asian patients, performed in the United States between 2004 and 2013. It was reported that Asian patients aged 15–44 years using autologous ART increased >10% over the 9 years, whereas non-Hispanic White women had an increase of <5%. Asian women had lower live birth rates with autologous ART (25.8%) than non-Hispanic White women (31.2%) (11).

Three studies conducted at their respective fertility centers also reported decreased clinical pregnancy and live birth rates. Langen et al. (12) studied 180 fresh blastocyst transfer IVF cycles (112 White and 68 Asian) from January 2005 to December 2006 at Stanford University Medical Center. Both the White and Asian groups were similar in treatment characteristics, the number of oocytes retrieved, fertilization rate, and the number of blastocysts transferred. Despite the similarities, Asian women had a lower implantation rate (28% vs. 45%, *P*=.01), clinical pregnancy rate (43% vs. 59%, *P*=.03), and live birth rate (31% vs. 48%, *P*=.02) (12). McQueen et al. (13) studied 4,045 women (3,003 White and 541 Asian women) who underwent their first autologous IVF cycle at Fertility Centers of Illinois from January 2010 to December 2012. The aOR of clinical pregnancy was 0.63 (95% CI, 0.44–0.88), and the aOR of live birth was 0.64 (95% CI, 0.51–0.80). However, in this study, the researchers also noted that Asian women required a longer duration of stimulation (10.3 days vs. 9.9 days, *P*=.00034), had a higher mean peak estradiol level (2,694.0 vs. 2,388.6, *P*<.0001), had fewer oocytes retrieved (12.1 vs. 13.6, *P*=.0043), and had fewer surplus blastocysts available for cryopreservation (1.5 vs. 2.0, *P*=.0004) (13). Shahine et al. (14) studied 225 patients (145 White and 80 Indian) who had a blastocyst transfer from January 2005 to June 2007 at Stanford University Medical Center. Indian patients had a significantly lower clinical pregnancy rate (36% vs. 52%, *P*=.02) and live birth rate (24% vs. 41%, *P*<.01). However, this study was limited by the small sample size and the inclusion of only Indian women (14).

In contrast, studies by Sharara et al. (15) and Gleicher et al. (16) found no difference in IVF outcomes for Asian women. The study by Sharara et al. (15) was limited by its small sample size of Asian women (n = 54), significant differences in baseline characteristics (including younger age and higher polycystic ovary syndrome [PCOS] diagnosis in the Asian cohort), and no documentation of whether these factors were controlled for in analyses. Gleicher et al. (16) conducted a single-center study analyzing 339 consecutive IVF patients for FMR1 genotypes and tested associations among race, FMR1 genotype, autoimmunity, and pregnancy outcomes with IVF. Their Asian sample size was also noted to be small (n = 48) (16).

Another study published by Lamb et al. (17) focused on the difference in intrauterine insemination (IUI) outcomes between Asians and their White counterparts at the University of California, San Francisco. The researchers performed a retrospective analysis of the cohorts of Asian and Caucasian patients treated with IUI between December 2002 and December 2006, including 2,327 IUI cycles among 814 patients. They concluded that the aOR of cumulative pregnancy rates after IUI was 0.68 (95% CI, 0.47–0.98) compared with White women. Of note, they also determined that a greater proportion of Asians (43.9% vs. 24.6%, *P*<.0001) presented for treatment with >2 years of infertility at the time of initial consultation, thereby suggesting that Asian patients tend to wait longer to seek infertility treatment (17).

Interestingly, a study published by Dieke et al. (18) investigated the data from the Centers for Disease Control and Prevention’s National ART Surveillance System to calculate the number of ART procedures per million women of reproductive age for each racial/ethnic category in 2014. It was reported that Asian and Pacific Islander women had the highest number of ART procedures per million women aged 15–44 years (5,883.0), followed by White non-Hispanic women (2,888.4) (18). Although Asian Americans have been reported to have worse ART outcomes, this study suggests a trend toward increased ART utilization.

The majority of published research on Asian American infertility shows that Asian women have greater difficulty conceiving and lower success rates of infertility treatment. There may be a myriad of explanations for this, including differing genetic susceptibilities, underlying infertility factors, and environmental factors. Each of these is explored further in this article.

## Genetic susceptibilities to infertility

Several genetic differences between Asian and White women have been suggested to contribute to disparities in ART outcomes. A higher number of FMR1 gene mutations have been found in Asian women with CGG repeats of >34. Typically, most women have 5–44 CGG repeats in their FMR1 genes; however, studies have shown that infertile women with a diminished ovarian reserve are more likely to have ≥35 FMR1 CGG repeats (19). A high number of FMR1 mutations has been associated with infertility and may thus contribute to these findings in Asian women (11).

**Figure 1:**
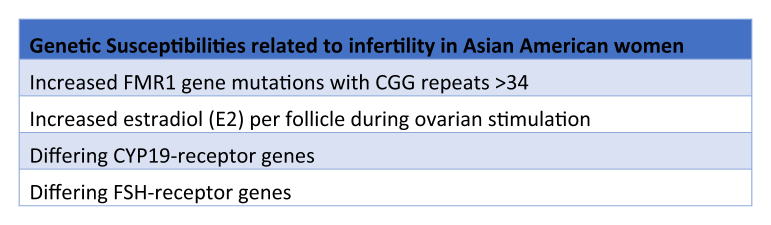
Asian American genetic susceptibilities to infertility. Multiple genetic differences between Asian and white women are suggested to contribute to disparities in assisted reproductive technology outcomes.

Although some studies have shown that Asian women have similar mean day 3 follicle-stimulating hormone levels, the number of follicles produced during stimulation, and the total number of oocytes retrieved and transferred (9), other studies have shown that there are higher exogenous follicle-stimulating hormone requirements during an IVF cycle in Asian patients who are homozygous for the serine variant in the follicle-stimulating hormone receptor gene (20). This may be due to the fact that homozygosity for the serine variant is associated with poor ovarian response and lower numbers of oocytes retrieved. Asian patients have also been shown to have allelic polymorphism frequencies in the CYP19 receptor genes compared with White patients. These polymorphisms are believed to affect estradiol synthesis and metabolism and thus contribute to different clinical outcomes (21, 22). A study conducted at a private reproductive medicine clinic in Australia of 2,594 patients (2,072 White and 522 Asian) undergoing IVF noted that despite requiring higher doses of gonadotropin, Asian patients achieved fewer oocytes and had resultant fewer embryos for transfer and cryopreservation (23). Figure 1 lists the genetic susceptibilities related to infertility in Asian American women.

## Underlying etiologies of infertility

Some common underlying etiologies of infertility in couples include endometriosis, ovulatory dysfunction, and male factor infertility. Studies have shown that there is a difference in the prevalence of these causative factors between Asian Americans and their White counterparts. Endometriosis is present in 9%–50% of infertile women (24). Asian Americans have significantly higher rates of endometriosis (15.7% vs. 5.8% for White patients, *P*<.05) (25). A prospective registry study in a Canadian tertiary referral center evaluating for endometriosis and pelvic pain found that, of the 1,594 women included, East and Southeast Asian patients (n = 145) were 8.3 times more likely than White patients (n = 1,214) to have a previous diagnosis of stage III/IV endometriosis before referral (95% CI, 3.74–18.57). A retrospective chart review of 717 patients undergoing their first round of IVF between 2008 and 2009 at the University of California, San Francisco, found that although Asian Americans have poorer ART outcomes, including clinical pregnancy rates, the discrepancy was conditionally independent on the presence of endometriosis (25).

Ovulatory dysfunction accounts for 15% of couples infertility (24). Polycystic ovary syndrome is one of the most common causes of ovulatory dysfunction, affecting approximately 8%–13% of reproductive-aged women (26). The prevalence of PCOS in Asian patients compared to White patients is limited and focused on specific ethnicities. The prevalence is 5.6% in Chinese patients and 5.7% in Thai patients based on the Rotterdam criteria vs. 4.9% in Korean patients based on the National Institute of Health criteria (27). Although White patients have been found to have a higher prevalence of PCOS, there is an increasing evidence that Asian women present with different phenotypic presentations and may have different fertility outcomes (28). For instance, South Asian women (typically defined as Indian or Asian-other) with PCOS have higher total mean Ferriman-Gallwey scores for hirsutism (29). East Asian women (typically defined as Chinese, Japanese, Korean, Filipino, Vietnamese, Thai, Nepalese, Cambodian, or Indonesian) with PCOS have increased central obesity (29). A study conducted in the United Kingdom comparing 324 IVF/intracytoplasmic sperm injection cycles between White (n = 220) and Asian (n = 104) patients diagnosed with PCOS found that White patients had a higher fertilization rate and a 2.5 times (95% CI, 1.25–5) higher chance of ongoing clinical pregnancy than their Asian counterparts (30).

Male factors account for 35% of couples infertility (24). There have been mixed findings regarding the semen quality of Asian patients compared with White patients. A study at Stanford University of 1,230 men presenting for semen analysis concluded that White patients had higher semen volumes than Asians (2.9 mL vs. 2.6 mL, *P*<.01); however, Asians had higher sperm concentrations (60.9 × 10^6^/mL vs. 51.3 × 10^6^/mL, *P*<.0001) (31). Another Canadian study of 3,956 infertile men from 2008 to 2017 found that Asian patients are more likely to have lower semen volumes (OR, 1.23; 95% CI, 1.01–1.51) and be azoospermic (OR, 1.34; 95% CI, 1.11–1.62) than White patients. The study also concluded that Asians had significantly lower testosterone levels in a linear regression analysis (−1.26 [−1.77– {−0.76}]), and this may be one of the reasons for the decreased reproductive parameters (32). Moreover, Asian men have been found to have higher rates of sexual dysfunction. A systematic review of 50 studies evaluating male sexual dysfunction in European and Asian populations from 2008 to 2018 found that the prevalence of erectile dysfunction, low satisfaction, and hypoactive sexual desire disorder was higher in Asian than European men (33).

Therefore, it is possible that various etiologies contribute to increased fertility issues in the Asian population. Additional research is needed to evaluate other factors, including unexplained infertility and tubal disease. It has also been speculated that body mass index may differentially affect IVF outcomes in certain races, including Asians (34).

## Environmental infertility factors

Environmental differences between Asian and White women may also contribute to Asian infertility. The National Health and Nutrition Examination Survey reported that Asian and Pacific Islanders had increased levels of methylmercury in their diet, a known reproductive toxin (35, 36). This is likely due to the fact that this toxin is seen more commonly in fish, and thus a seafood-heavy diet can contribute to this increased exposure. Among women of reproductive age, 83.1% of Asian patients reported consuming seafood in the past 30 days compared with 74.0% of non-Asian patients (*P*=.0043). Using the reported seafood consumption, the average daily mercury intake was estimated to be 0.03 μg/kg per day for Asian women of reproductive age and 0.02 μg/kg per day for their non-Asian counterparts (*P*<.0001) (35). Methylmercury has also been linked to an increased risk of spontaneous abortion. The exposure of cultured animal embryos to methylmercury has been shown to affect viability, suggesting similar effects on humans (15, 37).

## Psychosocial and cultural factors affecting infertility treatment

International and cross-cultural infertility studies have shown the detrimental effects that living in a highly pronatalist society can have on infertile couples, especially on women (38). A pronatalist society is the one that promotes the reproduction of human life (39). The United States, in particular, is a pronatalist country, where very low fertility has been recognized as a social problem requiring government action and policy changes (40). For instance, the US government recently approved the Child Tax Credit, which is a tax benefit to help families who are raising children (41).

**Figure 2:**
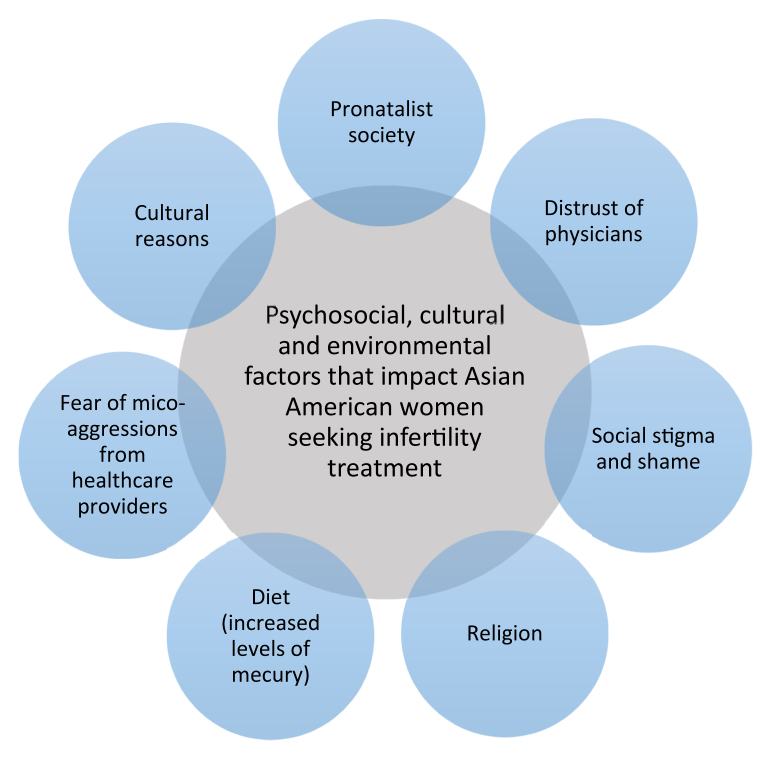
Psychosocial, cultural, and environmental barriers to seeking infertility treatment. Asian American patients face many barriers that affect their ability to receive infertility care.

Despite the growing rates of ART utilization, there is still evidence suggesting that Asian Americans are less likely to seek infertility care. Figure 2 shows the psychosocial, cultural, and environmental factors that affect Asian American women seeking infertility treatment. In Asian communities, women feel pressured to bear children and are frequently blamed for their inability to conceive. Asians facing infertility have been shown to experience extreme psychologic distress, including loss, anxiety, depression, feelings of hopelessness and loss of control, anger, resentment, and low life satisfaction (42, 43). Due to the social stigma and strong sense of shame, Asian women will tend to conceal their circumstances and are less likely to seek fertility treatment or seek treatment at a later age (3).

Distrust of physicians among Asian women also affects their desire to seek fertility care (44). Asian patients are more likely than White patients to report that their healthcare providers did not understand their backgrounds, involve them in healthcare decisions as much as they wanted, or treat them with respect (45). Additionally, common Asian microaggressions, including the belief that all Asians are well-educated and can speak and understand English fluently, can jeopardize the patient-provider relationship (46).

When Asian women seek fertility treatment, certain types of treatments, including gamete donation, either sperm or egg, are typically frowned upon in both religious and cultural contexts (47). When gamete donation is pursued, couples prefer Asian donors, and mixed ethnicity oocytes raise even more ethical dilemmas (48). Therefore, this limits the fertility treatments that Asian women feel comfortable pursuing.

## Limitations

The biggest limitation with the study of Asian American infertility is the use of the monolithic term “Asian” when collecting demographic data. According to the US Census Bureau, when a person checks “Asian” in the race category, they are “a person having origins in any of the original people of the Far East, Southeast Asia, or the Indian subcontinent, including, for example, Cambodia, China, India, Japan, Korea, Malaysia, Pakistan, the Philippine Islands, Thailand, and Vietnam” (5). This encompasses a broad range of ethnicities, each of which may have their own infertility patterns and trends. Asian Americans recorded the fastest population growth among all racial and ethnic groups in the United States between 2000 and 2019 from 10.5 × 10^6^ to 18.9 × 10^6^ (49). As the Asian American population continues to grow in record numbers, grouping all Asian patients together in fertility research can overgeneralize findings. This has been seen in other areas of obstetrics and gynecology. For instance, studies disaggregating data on Asian women to assess cancer screening noted that Filipino women had higher rates of breast and cervical cancer screening examinations than Indian, Korean, and Chinese women (50). Future studies could benefit from investigating the trends that differentiate racial and ethnic categories within the term “Asian,” such that we may tailor counseling and treatment among one group vs. another.

Furthermore, Asian American patients are less likely to participate in infertility research. Asians born within and outside the United States are less likely to express interest in being contacted about fertility research than non-Hispanic Whites (OR, 0.44; 95% CI, 0.33–0.58) (51) Many potential factors may affect their research participation, including the stigma surrounding infertility and distrust in their healthcare providers, as discussed in the abovementioned sections. There may be a language barrier when studies are conducted solely in English. Asian patients with strong religious beliefs have also been shown to be less likely to donate excess embryos for research (52). It is important that Asian patients be included in protocols for future infertility studies. Recruitment strategies such as using focus groups and the involvement of study personnel who speak the native language of patients may aid in overcoming participation barriers (38).

## Conclusion and recommendations

There are a limited number of studies focusing on the increased prevalence of Asian American infertility and decreased rates of treatment success. Additionally, research on the reasons behind the disparities in evaluation and treatment has only begun to emerge. It is suggested that genetic and environmental predispositions and cultural factors play a crucial role in Asian American infertility. Asian Americans may also have a higher prevalence of certain risk factors such as endometriosis and abnormal semen parameters.

To our knowledge, this is the first review article summarizing the current published literature on Asian American infertility, the potential etiologies, and the fertility treatment outcomes. It is important that we continue the research on factors affecting the access to, utilization of, and response to treatment. Recommendations for future research include updating data on Asian American infertility trends, differentiating between Asian racial and ethnic groups, and determining how to destigmatize participation in both qualitative and quantitative infertility research. This could aid in providing more comprehensive care to this important and diverse population.
